# Investigating the muti-scaling properties and connectedness of the sovereign bond yields: Hurst exponent and network analysis approach

**DOI:** 10.1016/j.heliyon.2023.e16666

**Published:** 2023-05-31

**Authors:** Santanu Das, Aritra Pan, Nikunj Kumar Jain

**Affiliations:** a Finance & Economics, International Management Institute, IDCO Plot No. 1, Gothapatna, Chandaka Malipada, Bhubaneswar, Odisha 751003, India; b Information Management & Analytics, International Management Institute, IDCO Plot No. 1, Gothapatna, Chandaka Malipada, Bhubaneswar, Odisha 751003, India; c Production and Operations Management, Indian Institute of Management Nagpur, Plot No. 1, Sector 20, MIHAN (Non-SEZ), Nagpur, 441108, India

**Keywords:** Multi-scaling, Sovereign bonds, Hurst exponents, Spectra analysis, Regularized network modelling

## Abstract

Using daily yield data of 14 sovereign bond markets from emerging and developed economies from July 10, 2000, to July 10, 2022, we examine their scaling properties using generalized Hurst exponent and spectral density analysis and investigate the connectedness based on a network analysis approach. We consider the yields of 2-year and 10-year bond yields to investigate the scaling properties for short- and long-term sovereign bonds. This selection also allows us to examine sovereign bond spreads with respect to the USA. We also use regularized partial correlation network analysis to connect different countries in communities based on yields. We find that the scaling behavior of the bond yields for both terms fits well using the Hurst exponent and spectral analysis confirms this finding. Moreover, we also find that even though bonds in both cohorts show anti-persistent behavior except that of the USA, the developed economies' bond yields are relatively less anti-persistent as compared to those of emerging economies. The networks of both the 2-year and 10-year yields indicate community formation in various countries which provides diversification benefits to the investors. Most of the emerging countries are classified into one community in the long-tenure bonds as well but this concentration is more evident in the short-tenure bonds.

## 1 Introduction

The concept of scaling, which originates in the discipline of science, particularly physics is increasingly getting popular in economics and finance as well, and accordingly, a new stream of research has evolved – econophysics. The scaling property in time series refers to the patterns in financial asset prices that are repeated at different time scales. Researchers in finance have always been interested in identifying the pattern in asset prices, especially in high frequency. This was initiated by Elliot in the 1930s, where he emphasized the repetition of patterns in asset prices. This was followed by the application of the Brownian motion in understanding the scaling of asset prices. Under this, changes in asset prices are assumed to be normally distributed with zero mean and finite variance. Studies in asset prices provide ample evidence that returns are not normally distributed and often they show heavy tails (high kurtosis) [1,2,3,4]. In an interesting study, Matteo et al. [5] found that deviations from Brownian motions depend upon the degree of development of financial markets. Using the generalized *Hurst exponent*, which measures the long-term persistence of a time series, he shows that different sub-periods reveal different scaling behavior of a given market. Although the scaling techniques used so far are not free from criticism, they show a remarkable improvement over conventional techniques to describe the behavior of asset prices in the time-frequency domain.

In the past one and a half decades or so, there has been tremendous development in the application of fractals and scaling techniques to understand the dynamics of financial time series data. This has been made possible to a large extent due to the availability of asset price data at various frequencies. Financial data are characterized by a probability distribution that cannot be explained by Gaussian distribution and studies have shown that they follow the power law behavior [6]. Although various techniques have been used to study this non-Gaussian distribution, such as the GARCH family models [7,3], and leptokurtic distribution using a mixture method [8], these have not been able to capture the scaling properties of the data distribution. Some studies provide evidence that financial time series data exhibit certain scaling behavior [9,10]. These studies have provided new insights into the behavior of time series data, and it is therefore important that we investigate the scaling properties in one of the most important asset markets of the world – the sovereign bond market. Sovereign bonds are a widely studied asset class, but methodologies related to measuring risk or persistence is rather limited [11]. In yet another study, Zunino et al. [12] study the complexity-entropy causality plane in order to unveil the presence of correlations and hidden structures in the daily values of thirty bond indices of both developed and emerging economies. In our paper, we unearth the long-term persistence of the sovereign bond yields, which is methodologically different from Zunino et al. [12] who focus of the correlations among the permutation entropy, economic development, and market size.

In this paper, we use scaling methods to detect the underlying regularities in sovereign bond markets. Past studies have studied the scaling behavior in equity markets [9,13,5] and the foreign exchange market [14], but no such effort has been made to the best of our knowledge to study the scaling and fractal behavior in the sovereign bond market. Sovereign bond yields and their spreads indicate the risk premia of a given country, which in turn affects the investor's appetite for investment in sovereign bond markets. The behavior of these bonds, especially during and after a crisis warrants an investigation which may guide the policymakers as well as investors to take informed decisions [15]. Moreover, the yields, especially the long-term also provide guidance for the risk-free rate essential for asset pricing. We find that the Hurst exponent fits very well into the yield data of sovereign bonds which are confirmed using the spectral density analysis. We also find that on average, the bond markets of the developed economies are less anti-persistent compared to those in emerging economies. Our study also uses regularized network analysis to investigate the correlation among yields. In a recent paper, Miyakoshi and Shimada [16] assess the correlation in the local currency bond yields in nine Asian markets - Indonesia, Korea, Malaysia, Thailand, China, Hong Kong, the Philippines, Singapore, and Japan using network analysis. They argue that network analysis focuses on the market structure of the region using the network indicators above and is ideal for investigating these problems. Similarly, network analysis has also been used in studies in the context of banking data from developed countries [17,18].

Therefore, we contribute to the extant literature in three ways. First, we use the multi-scaling technique to understand the underlying time series behavior in time-frequency domain of the sovereign bonds of emerging and developed economies. Second, our study shows that the time series properties in time-frequency domain for the long term and short rem bonds are different thereby underlining the importance of persistence of the yields of different tenure bonds which vary over time. Finally, we contribute methodologically by using an optimized network to investigate the correlation among the bond yields.

## 2 Objectives of the study

Considering the importance of understanding the interconnectedness and persistence behavior of sovereign bonds, we outline three objectives of this study.

(i) To study the long-term persistence of yields of the long-term and short-term sovereign bonds.
(ii) To introduce the use of novel methodologies of Hurst exponent and network analysis for understanding the relationship among the sovereign bonds.
(iii) To understand the dynamics of correlation of the bond yields in developed and emerging economies.

## 3 Data methodology

### 3.1 Data

We collect daily yields of 2-year and 10-year sovereign bonds of 14 countries – USA, UK, Germany, France, Austria, Japan, Spain, Poland, Peru, China, India, Brazil, Malaysia, and Thailand. All yields are measured in percentage. The sample is further classified into developed (USA, UK, Germany, France, Japan, Spain, Poland, and Austria, we call it Cohort 1) and emerging (Peru, China, India, Brazil, Malaysia, and Thailand, which we call it Cohort 2) countries. The sample period ranges from July 2000 to July 2022. All the data have been collected from Bloomberg.

### 3.2 Methodology

In this paper, we use Hurst exponent to identify the bonds of both long-term and short-term tenure for their long-term persistency. Based on the results, we create communities of countries using regularized partial correlation networks from yields. The graphical LASSO (least absolute shrinkage and selection operator) network structures used in this study is different from other networks in terms of representing directional correlations as the edge between two nodes or countries. The overall networks are optimized by using a LASSO penalty (***λ***) value to reduce the number of spurious edges as well as balancing it with stricter network with minimum number of edges. Therefore, the networks are balanced for maximum specificity without losing valuable information over the edges.

#### 3.2.1 Hurst exponent

We use the Hurst exponent proposed by Hurst [19] to explain the long-term dependency on reservoir water flow. In the context of financial time series, it measures the long-term memory and fractality of a time series. A Hurst value equal to 0.5 indicates a Brownian process, a value more than 0.5 indicates trending or momentum and a value less than 0.5 indicates a mean reverting process. Although there are many techniques to study the scaling properties like the rescaled range analysis (R/S analysis), detrended fluctuation analysis (DFA), or generalized Hurst exponent (GHE), Matteo [14] suggests the use of the generalized Hurst exponent (represented as H(q)) to study the scaling structures of different financial time series because unlike the R/S or DFA it is not affected by the presence of outliers in the data. He finds that GHE provides an unbiased and computationally efficient estimator able to explain the scaling features of financial time series data. Ho et al. [13] confirm the existence of multi-fractal properties in the Taiwan stock exchange and the GHE can capture this property well. In a recent study, Ferreira [20] using the Hurst exponent finds long memory in the sovereign yields of the Euro zone.

We first define a time series *X(t)*, *t = 1ν, 2ν, …,kν, T* with the observation period *T* and time-resolution *ν*. This kind of scaling can be explained by an exponent *H(q)* which is associated with long-term persistence, where *q* is the moment of the distribution. To analyze *q-order* moments of the distribution, we define:

(1) $$K_{q}\left(\tau \right)=\frac{{\left|X\left(t+\tau \right)-X\left(t\right)\right|}^{q}}{{\left|X\left(t\right)\right|}^{q}}$$

for $\tau \;\varepsilon \;\left(1,\tau_{\left(m_{ax}\right)}\right)$ and $\tau_{\left(m_{ax}\right)}$ are usually chosen as a quarter of the length of the series.

Note that in equation (1) above, if *q = 2*, it is proportional to the autocorrelation function. For *q* *=* 2 which corresponds to the autocorrelation function of the increments is crucial for understanding long-term dependencies [21,22].

The GHE can now be defined from the scaling behavior of $K_{q}\left(\tau \right)$ as:

(2) $$K_{q}\left(\tau \right)\;∼\;{\left(\frac{\tau }{\nu }\right)}^{qH\left(q\right)}$$

If *H(q) = H*, a constant which is independent of *q* is a feature of uni-scaling that coincides with the original Hurst exponent (for *H =* 0.5, it indicates the Brownian process). If, however, *H(q)* depends upon *q*, then the process is multi-scaling [23,14,24].

The GHE technique is quite appealing because all the information about the scaling properties of the time series is reflected in only one measure, *H(q)*. The Hurst exponent is characterized by the value *q* takes. For example, *q = 1*, signifies the scaling properties of absolute deviation of the time series. In this study, we consider *q = 2* for investigating the long-term dependencies of the sovereign bond yields.

#### 3.2.2 Multi – scaling in the time-frequency domain

To examine the statistical properties of the sovereign bond yields, we conduct the spectral density analysis in the time-frequency domain. We use the power spectral density method of Kay and Marple [25]. The spectral density *S(f)* of many financial time series have been shown to obey the power law as *S(f)* $\propto f^{-\beta }$ , where β measures the slope of the power spectrum. This slope is related to the GHE for *q = 2* through *β = 1* + *2H(2)*. The autocorrelation function of the increments of the yields is proportional to the second moment of the distribution and then equation (2) will scale as *K*_*2*_ ∼ $\tau^{2H\left(2\right)}$. In a Fourier transform, a function that shows the time domain as $\tau^{m}$ is proportional to *f*^*-m−1*^ in the frequency domain. Therefore, the spectral of a time series is shown as:

(3) $$S\left(f\right)\propto f^{-2H\left(2\right)-1}$$

The behavior of the power spectra *S(f)*, which is computed from equation (3), is then compared with the function *f* ^*−2H(2)−1*^which is the scaling behavior in the frequency domain with the GHE *H(2)*. Finally, the slope of the spectra *β* is associated with the GHE as show in equation (4):

(4) β = 1+2H(2)

#### 3.2.3 Network estimations

Nagurney and Ke [26] proposed a framework to analyze the financial network equilibrium problems with intermediation and variable weights. Barro and Basso [27] investigated the credit risk of a bank loan portfolios using a dynamic framework. Capponin and Chen [28] showed that the interconnection between financial markets and institutions across countries can be captured by a network structure. Sensoy et al. [29] used a correlation-based network to understand the financial integration and segmentation of European Monetary Union (EMU) sovereign bond markets based on the network structure. Fiscal performance was found to be the primary reason behind segmentation.

This paper introduces regularized partial correlation network models in economics and finance domain to estimate the relationships between daily yields of different countries. We have developed two separate networks to understand the connectedness between daily yields of 2 year and 10 years sovereign bonds. This methodology is unique since it is a popular and widely used methodology to understand connectedness between different factors in psychological networks [30] and has never been introduced to the domain of finance before. This paper uses partial correlation instead of pearson's correlation coefficient which only reveals linear relationships and ignores the heterogeneity of financial data and cannot accurately measure the tail correlation [31]. This study attempts to utilize this robust method to estimate the connectedness of daily yields. Edges in PMRF networks are the relationship between each pair of countries after controlling for their shared variance with all other countries in the network (*i.e.* conditionally dependent relationships). These are the “state-of-the-art” in psychopathology network modelling and have become popular and widely used [32]. Regularisation-based model selection uses LASSO [33] with a hyperparameter tuning to minimize the Extended Bayesian Information Criterion (EBIC) [34]. This technique limits the total sum of absolute edge weights, thus shrinking many estimates to zero to exclude them from the network which resulted in a sparse network having high specificity by excluding spurious edges and identifying only “true” country – to – country relationships [35,30,36]. In small sample sizes like used in this study, using LASSO regularisation with EBIC for model selection is a conservative approach to network estimation because even moderately large edge weights may be set to zero, which may increase false negatives (i.e., “true” edges may not be estimated), but is intended to maximize specificity for avoiding any false positives. This paper performed regularized model selection using Least Absolute Shrinkage and Selection Operator (Graphical LASSO) using a package called “qgraph” in R statistical software [37].

In networks, countries are nodes and partial correlation of yields between two countries is an edge. Thickness and saturation in edges represent their magnitude respectively. Green edges indicate positive correlation value and red edged indicate negative correlation value [37]. “Glasso” helps to identify relevant edges and efficiently discover underlying network structure through a maximum likelihood solution by minimizing an Extended Bayesian Information Criterion (EBIC) with help of tuning parameter (λ). This paper uses graphical LASSO (least absolute shrinkage and selection operator) in combination with EBIC model selection [38] for network modelling of pairwise Markov random field (PMRF) networks. λ for the “glasso” estimation can be kept between 0 and 0.5 for optimization of the model fit as well as parsimony [34]. Setting 0 would give more connections but will also bring in more spurious connections, where setting the value at 0.5 means more parsimony, but in cost of missing more connections [39]. The LASSO tuning parameter or penalty lambda (λ) value is taken as 0.25 to result in a network structure that minimizes the number of spurious connections while maximizing the number of true connections [38,40] by keeping a balance between true positives and false positives in edges. The graphical representations of network models are based on force-directed placement algorithm by Fruchterman and Reingold [41]. This algorithm puts stronger or highly connected nodes preferably at centre of the network, whereas lesser strong or connected nodes in the periphery.

Tabak et al. [42] developed an interpretation for systemic risk measures through clustering coefficients by evaluating different clustering coefficients and showed that the directed clustering coefficient may be used as a measure of systemic risk in complex networks. However, this paper deals with undirected graphs. Hence, a community detection algorithm was applied on the networks to detect communities in the respective networks. This paper uses spin-glass algorithm which is a modularity-based community detection algorithm and tests for communities in the network where by the number and weighted strength of edges within a cluster exceed the number and weighted strength of edges between nodes in another cluster [43]. Spinglass is robust in performance [44]. For small enough networks (N ≤ 1000) Spinglass outperforms other algorithms [45]. As a result, LASSO regularized network was used along with spin-glass algorithm.

## 4 Results and discussion

### 4.1 Hurst exponent

In this section, we discuss the results of the GHE and the spectral density plots. In Table 1, we report the descriptive statistics of 2-Year sovereign bonds. We find that the median yields in cohort 1 are conspicuously lower than those in cohort 2. The least median yield in cohort 1 is that of Germany (0.652%) as compared to the least median of Thailand (2.10%) in cohort 2, The average spread of yields of cohort 2 is about 357 basis points with reference to the yields of the USA. This shows that investors demand a high premium for investing in emerging countries.

**Table 1:** Descriptive Statistics of 2 Year daily yields (in % except observations).

Country	Mean	Median	1st Quartile	3rd Quartile	Standard Deviation	Maximum	Minimum	Observations
**Developed Countries**								
USA	1.839	1.386	0.519	2.748	1.563	6.455	0.1013	5502
UK	2.126	0.867	0.453	4.372	2.032	6.073	−0.16	5560
Germany	1.259	0.652	−0.551	2.72	1.827	5.3	−1.014	5622
France	1.357	0.951	−0.425	2.741	1.799	5.352	−0.837	5630
Japan	0.132	0.09	−0.117	0.182	0.297	1.088	−0.366	5380
Spain	1.834	2.13	−0.124	3.334	1.772	6.571	−0.694	5629
Poland	4.569	4.555	1.738	5.569	3.615	18.78	−0.039	5507
Austria	1.363	0.919	−0.475	2.795	1.845	5.442	−0.865	5578
**Emerging Countries**								
Peru	4.134	4.06	3.004	5.392	1.509	7.014	0.773	1627
China	2.730	2.694	2.248	3.234	0.705	4.384	1.125	3212
India	6.717	6.764	5.766	7.736	1.421	10.933	3.847	4377
Malaysia	4.231	4.261	3.997	4.554	0.392	5.194	3.055	3208
Brazil	9.736	9.893	7.684	12.290	3.051	16.834	3.223	2943
Thailand	2.273	2.107	1.494	2.995	1.079	5.31	0.341	4944

In Table 2, the descriptive results of the 10-year yields are shown. A similar pattern emerges for 10-year yields. However, the sovereign spread in long-term bonds is about 295 basis points which is less than the shorter leg of the spread. This indicates investors are more concerned about the short-term uncertainties of the emerging countries.

**Table 2:** Descriptive Statistics of 10 Year daily yields (in % except observations).

Country	Mean	Median	1st Quartile	3rd Quartile	Standard Deviation	Maximum	Minimum	Observations
**Developed Countries**								
USA	3.122	2.92	2.076	4.201	1.271	6.154	0.506	5502
UK	2.969	2.963	1.455	4.584	1.624	5.548	0.075	5559
Germany	2.254	2.297	0.412	3.991	1.844	5.341	−0.858	5629
France	2.556	3.005	0.784	4.058	1.737	5.499	−0.439	5630
Japan	0.839	0.947	0.094	1.39	0.642	1.999	−0.295	5366
Spain	3.272	3.888	1.548	4.530	1.747	7.566	−0.019	5629
Poland	4.896	5.075	3.150	5.956	2.374	13.664	1.135	5301
Austria	4.087	4.319	2.679	6.786	1.680	6.786	0.611	5518
**Emerging Countries**								
Peru	5.749	5.728	5.209	6.288	1.081	10.045	3.244	3542
China	3.468	3.436	3.102	3.771	0.488	4.711	2.472	3715
India	7.446	7.506	6.593	8.063	1.184	11.764	4.953	5343
Malaysia	3.888	3.899	3.583	4.138	0.530	5.937	2.398	4477
Brazil	10.918	11.14	9.598	12.365	2.086	16.839	6.396	2812
Thailand	3.513	3.512	2.494	4.544	1.302	6.692	0.810	4873

Further, we evaluate the spreads during the recent Covid-19 pandemic as well for the short and long-duration yields. The spread of the cohort 2 countries over the USA is about 321 basis points for the longer leg of the yields and it is 338 basis points for the shorter leg of the yields. The spread of the bonds is high during the crisis period indicating the overall pessimism in the bond market.

**Fig. 1:**
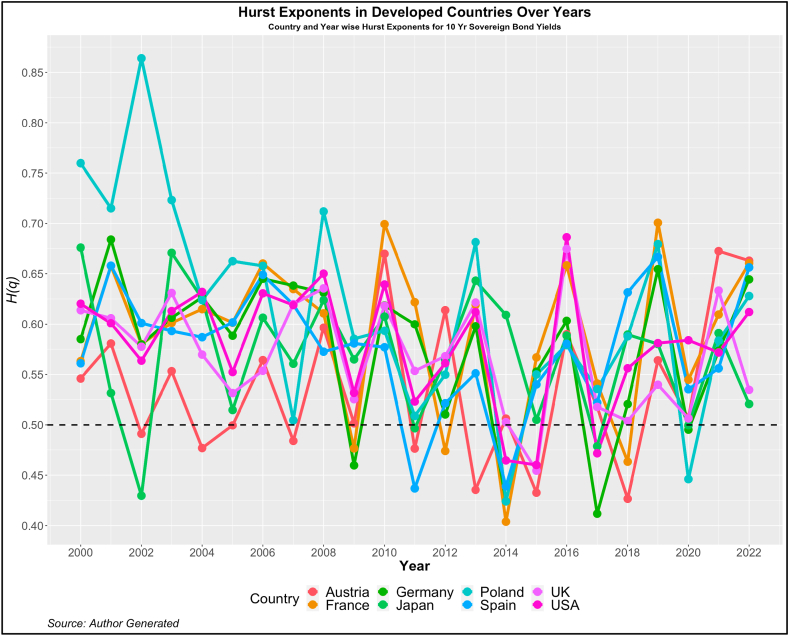
Year-wise GHE (10-year Sovereign bond yields) in Developed Countries.

**Fig. 2:**
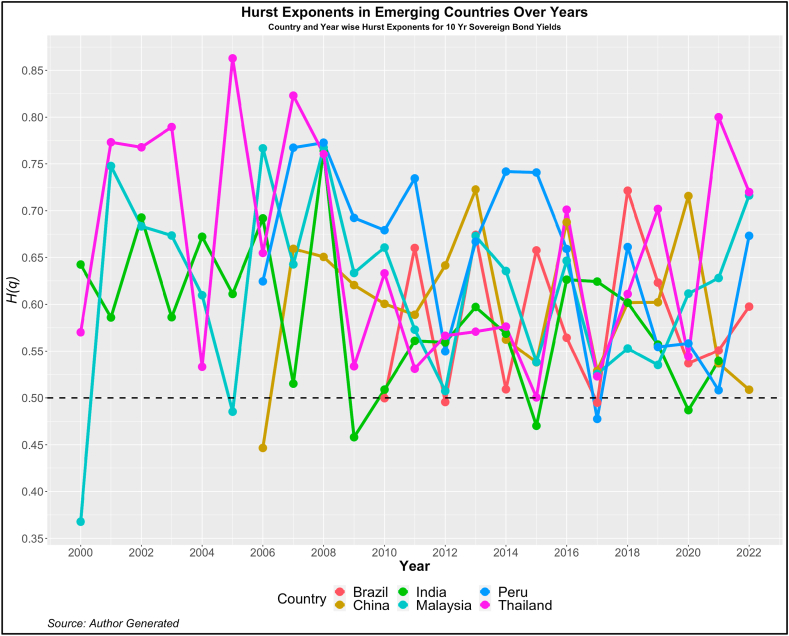
Year-wise GHE (10-year Sovereign bond yields) in Emerging Countries.

**Fig. 3:**
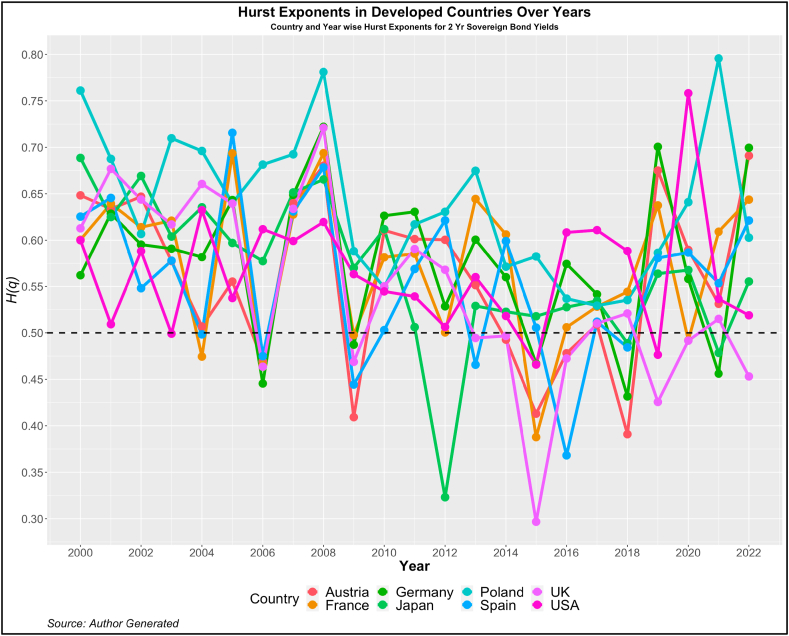
Year-wise GHE (2-year Sovereign bond yields) in Developed Countries.

**Fig. 4:**
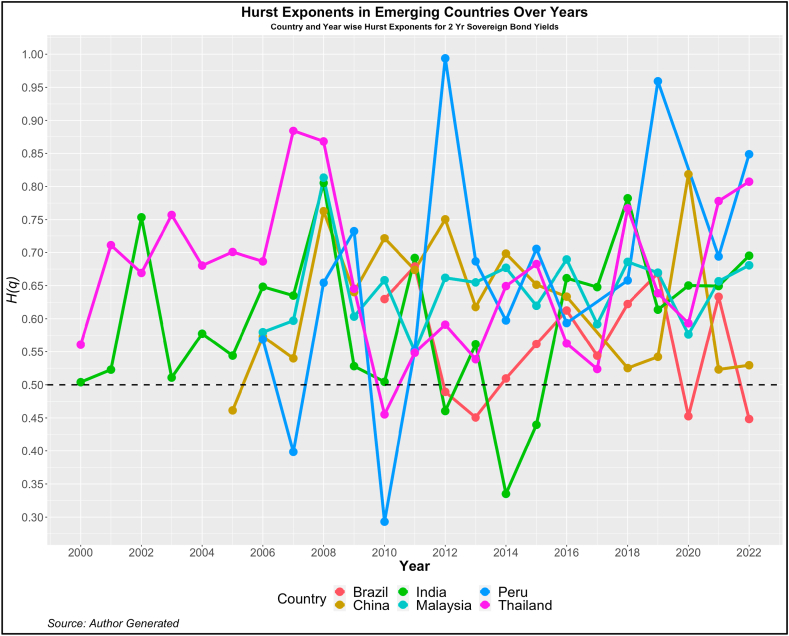
Year-wise GHE (2-year Sovereign bond yields) in Emerging Countries.

Now, we turn our attention to investigating the dependency pattern of the yields using the Hurst exponents. In Fig. 1, Fig. 2, Fig. 3, Fig. 4, we present the values of ***H(2)*** of 10-year and 2-year yields respectively for developed and emerging economies over the entire sample period. The dotted line indicates the value of ***H(2)*** *=* *0.5* which shows the random walk.

Values above this are trend or anti-persistent and values below indicate mean reversion. We see that as compared to cohort 1, cohort 2 is highly anti-persistent. This indicates that the impact of uncertainties in the 10-year yields is high which pushes up the yields resulting in the trend behavior. Another noticeable pattern is the values of *H(q)* in both developed and emerging economies were much higher than 0.5 during the crisis period (2008, 2009, 2010, 2019, and 2021).

In Table 3, Table 4, Table 5, Table 6 represent the summary of Hurst exponent values for each selected country for 10-year and 2-years yields respectively. The mean GHE of the 10-year yields of both developed and emerging markets (Table 3, Table 4) are above 0.5 indicating high degree of anti-persistence. However, emerging market yields show a higher standard deviation as compared to that in developed markets. It shows the wide variability in the GHEs over the sample period. Further, the minimum GHE in emerging markets is also less than the developed markets indication a faster mean reversion of the yields. The lowest GHE of 10-year yields among the emerging markets are reported for Malaysia and among developed markets for France.

**Table 3:** Descriptive Statistics of GHE of 10 Year yields in Developed Countries.

Country	Mean	Median	1st Quartile	3rd Quartile	Standard Deviation	Maximum	Minimum
Austria	0.538	0.518	0.487	0.585	0.073	0.672	0.427
France	0.59	0.61	0.554	0.646	0.077	0.701	0.404
Germany	0.576	0.598	0.537	0.629	0.074	0.684	0.412
Japan	0.569	0.58	0.518	0.608	0.063	0.676	0.43
Poland	0.615	0.593	0.55	0.68	0.103	0.864	0.424
Spain	0.576	0.58	0.546	0.611	0.061	0.667	0.437
UK	0.569	0.568	0.529	0.619	0.056	0.675	0.454
USA	0.58	0.584	0.554	0.62	0.06	0.686	0.46

**Table 4:** Descriptive Statistics of GHE of 10 Year yields in Emerging Countries.

Country	Mean	Median	1st Quartile	3rd Quartile	Standard Deviation	Maximum	Minimum
Brazil	0.584	0.564	0.509	0.658	0.078	0.721	0.495
China	0.601	0.602	0.538	0.65	0.075	0.723	0.447
India	0.587	0.586	0.544	0.626	0.077	0.764	0.458
Malaysia	0.617	0.633	0.545	0.673	0.097	0.768	0.368
Peru	0.651	0.667	0.558	0.734	0.092	0.773	0.478
Thailand	0.654	0.633	0.555	0.764	0.114	0.863	0.501

**Table 5:** Descriptive Statistics of GHE of 2 Year yields in Developed Countries.

Country	Mean	Median	1st Quartile	3rd Quartile	Standard Deviation	Maximum	Minimum
Austria	0.561	0.577	0.5	0.637	0.09	0.691	0.391
France	0.574	0.6	0.503	0.633	0.079	0.694	0.388
Germany	0.577	0.582	0.535	0.63	0.082	0.722	0.432
Japan	0.566	0.568	0.525	0.618	0.08	0.689	0.323
Poland	0.639	0.631	0.584	0.69	0.078	0.796	0.529
Spain	0.557	0.569	0.501	0.621	0.082	0.716	0.368
UK	0.544	0.521	0.482	0.625	0.098	0.721	0.297
USA	0.565	0.56	0.519	0.604	0.064	0.758	0.466

**Table 6:** Descriptive Statistics of GHE of 2 Year yields in Emerging Countries.

Country	Mean	Median	1st Quartile	3rd Quartile	Standard Deviation	Maximum	Minimum
Brazil	0.562	0.562	0.489	0.63	0.085	0.679	0.448
China	0.627	0.633	0.54	0.698	0.101	0.818	0.461
India	0.597	0.614	0.517	0.656	0.115	0.805	0.335
Malaysia	0.645	0.657	0.597	0.677	0.062	0.813	0.551
Peru	0.662	0.658	0.581	0.719	0.185	0.994	0.293
Thailand	0.665	0.669	0.577	0.734	0.111	0.884	0.455

Similarly, on examining the GHEs of 2-year yields, we find a similar pattern as that in the 10-year yields. The yields on an average exhibit anti-persistent behavior in both the developed and emerging markets. The yields of the UK and Peru among developed and emerging markets respectively, show a high standard deviation indicating high variability in the GHEs. This is further substantiated by the minimum GHEs reported for these two countries. The UK has a minimum GHE of 0.297 and that of Peru is 0.293. In fact the variability is very high for the yields of Peru with maximum GHE of 0.99. Therefore, although the markets show a high anti-persistent behavior, the variability in the GHEs should be considered by the investors before investing.

**Fig. 5:**
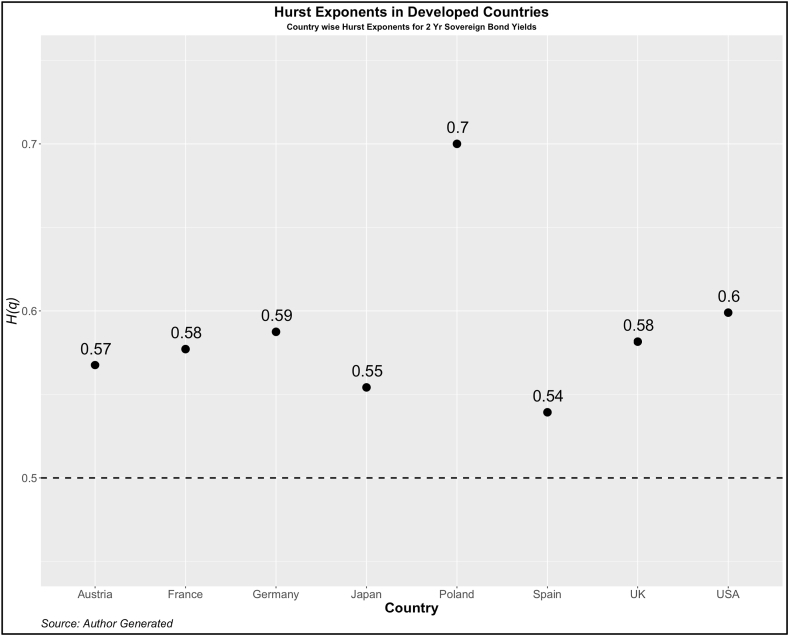
Country-wise GHE (2-year Sovereign bond yields) in Developed Countries.

**Fig. 6:**
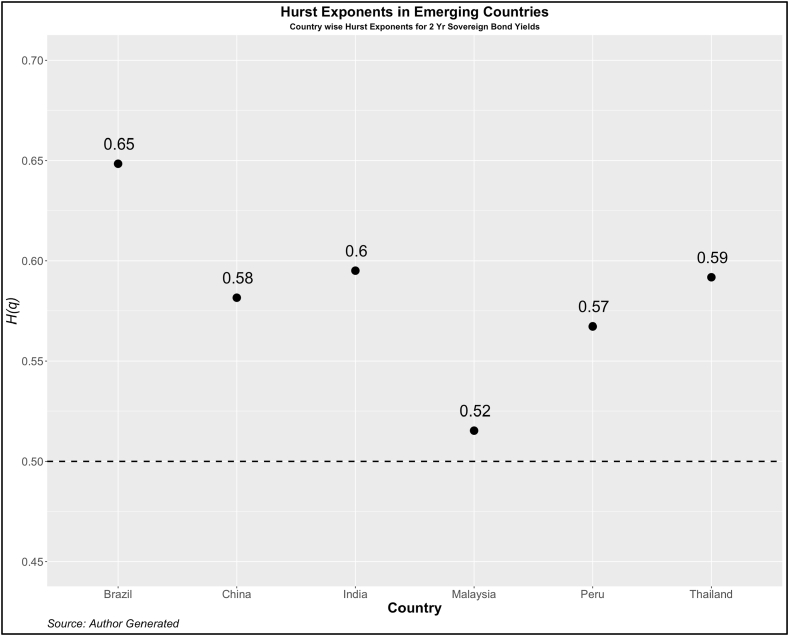
Country-wise GHE (2-year Sovereign bond yields) in Emerging Countries.

**Fig. 7:**
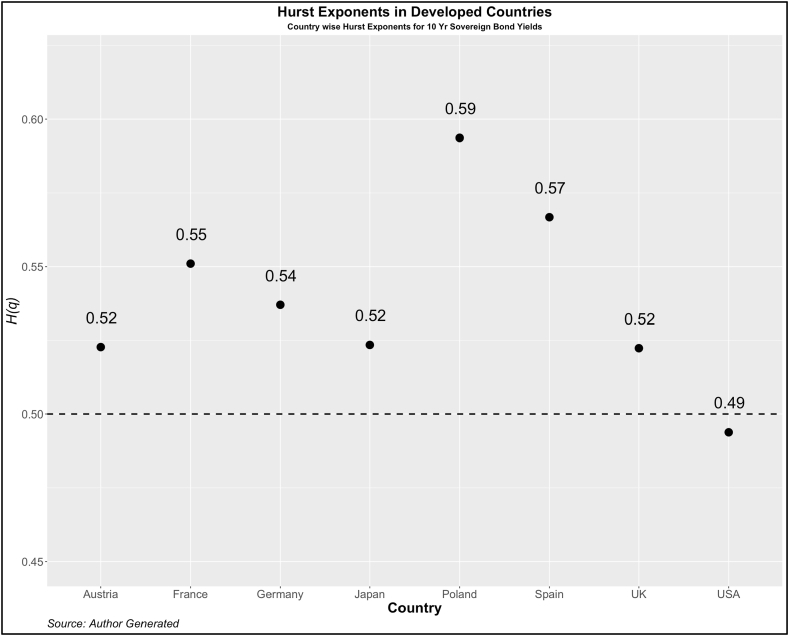
Country-wise GHE (10-year Sovereign bond yields) in Developed Countries.

**Fig. 8:**
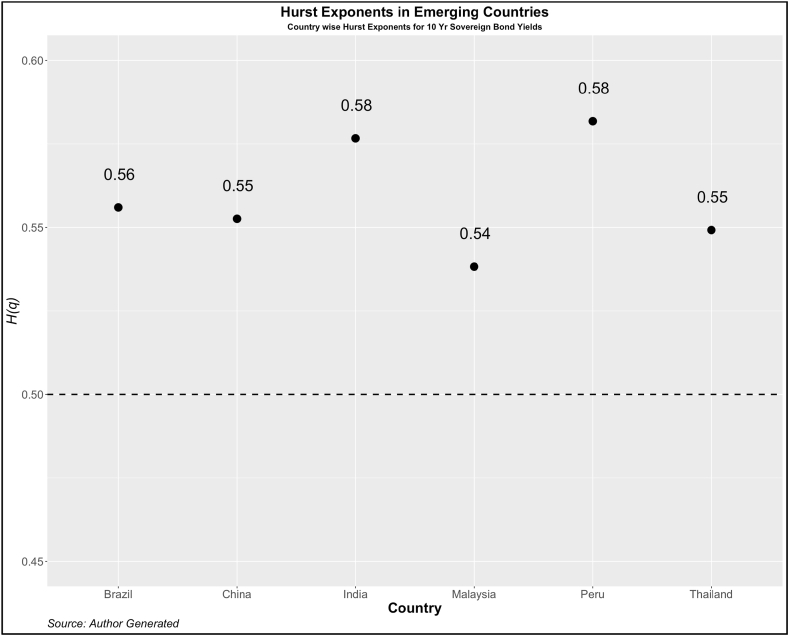
Country-wise GHE (10-year Sovereign bond yields) in Emerging Countries.

To further investigate the pattern of *H(q)*, we investigate the GHE for each country. The results are reported in Fig. 5, Fig. 6 (Cohort 1 and 2) for 2-yr sovereign bonds and Fig. 7, Fig. 8 (Cohort 1 and 2) for 10-yr sovereign bonds.

As expected, the GHEs of developed economies are lower than those of developed countries. Among cohort 1, the 10-year bond yields of the USA show a mean reverting property. The results contrast with Matteo et al. [5] who in the context of stock markets, find that the GHE of a few developed countries (Japan, USA, France, UK, and Germany) is less than 0.5 but those of Poland, Jakarta, Malaysia, Taiwan have the GHE of more than 0.5. This categorization is difficult to find using any other method and therefore we emphasize the importance of GHE in examining the scaling properties of various other asset returns.

### 4.2 Network estimations and modelling

**Fig. 9:**
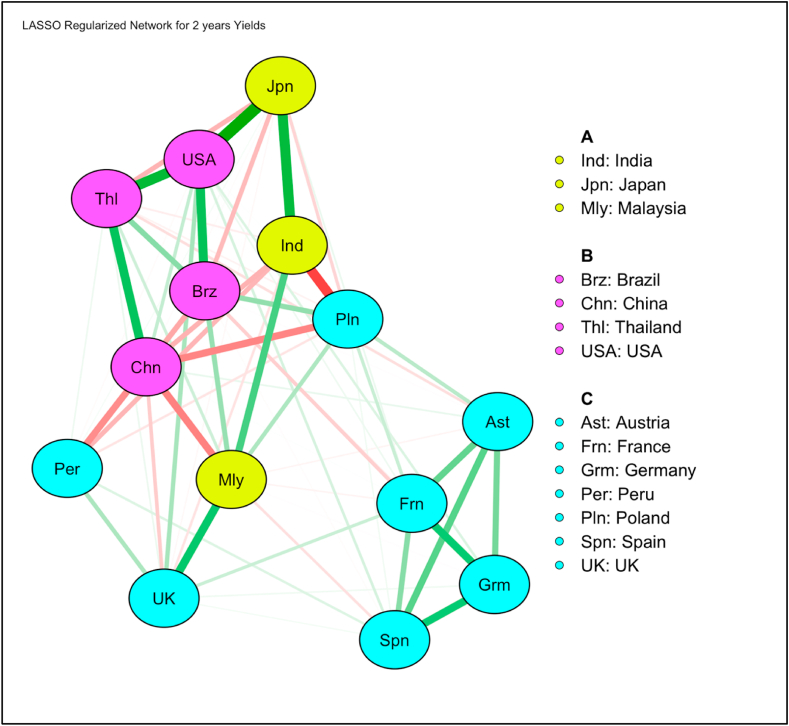
Estimated Network of Partial Correlations on 2 years Yields showing 3 communities.

In Fig. 9, Fig. 11, we have plotted the estimated networks for the yields of 2 years and 10 years respectively using regularized partial correlation networks (graphical LASSO network structures). Network structure models do not need “to carve nature at its joints” [46]. In Fig. 9, we find a total of 3 communities across all the countries selected in this study. In community 1, India has strong positive correlations with Japan and Malaysia. In community 2, Thailand has strong positive correlations with rest of the three countries i.e., USA, China, and Brazil. However, China and Brazil have negative correlation. China and USA have comparatively weaker positive correlation. Apart from these, there are few other significant observations like India and Poland have very strong negative correlation. Poland also has strong negative correlation with China. On the other hand, China has strong negative correlation with Peru and Malaysia. However, UK found to be in strong positive correlation with Malaysia. Japan has comparatively weaker negative correlation with Thailand, Brazil and Poland. These results indicate that countries in a particular community offers no diversification benefit to the investors. Further, we also find that the biggest benefit in international diversification in the 2-year bonds exists among emerging markets, notably among India, Poland, China and Malaysia and Brazil (see the red colored edges of the nodes, which indicate negative correlations). China and Brazil offer some diversification benefits even though they are in the same community.

In Fig. 11, we can see a total of 5 communities to be present in network structure of 10-year yields. France and Germany who are in very strong positive correlation form one community. Similarly, Thailand and Brazil also form a separate community having strong positive correlation. We found that community structures and members are significantly different in the case of 10-year yields as compared to 2-year yields. For example, India and Poland are in strong positive correlation here. Spain and UK have negative correlation which is opposite from very weak positive correlation found in 2-year yields. Poland and Austria are in negative correlation which is also opposite from the findings in 2-year yields. The investors from Poland – Austria, USA – Spain, UK – Spain, Japan – Poland and India – UK can get diversification benefits by investing in these bonds. Most of the emerging countries are classified into one community in the long tenure bonds as well but this concentration is more evident in the short tenure bonds. This suggests that while diversification can be achieved in 10-year bonds, they are less prominent in the 2-year bonds.

**Fig. 10:**
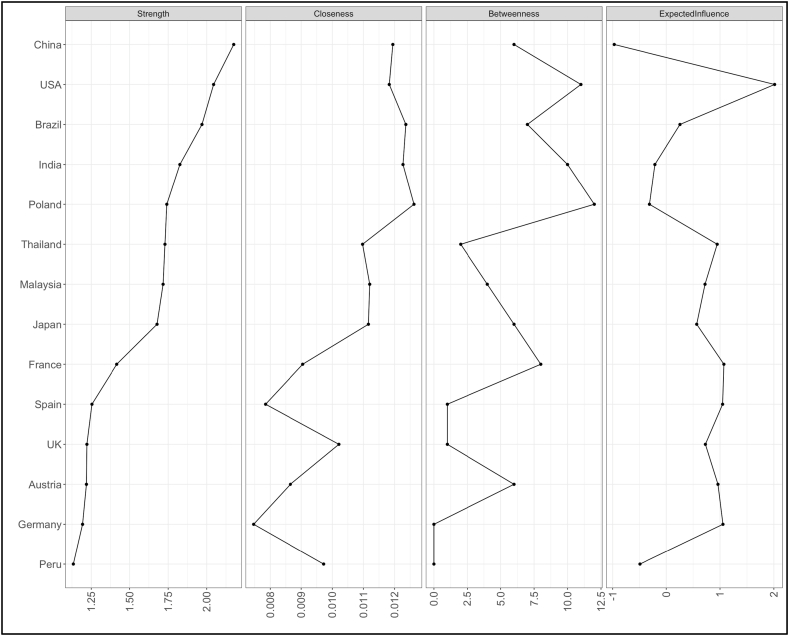
Centrality of 2 years yields network.

Fig. 10, Fig. 12 represent node centrality in 2-year and 10-year yields networks respectively [47]. For each node, we calculated strength (absolute sum of edge weights connected to a node), closeness (average distance from the node to all other nodes in the network), betweenness (the number of times that a node lies on the shortest path between two other nodes) [39] and expected influence (sum of all edges extending from a given node where the sign of each edge is maintained) [48].

**Fig. 11:**
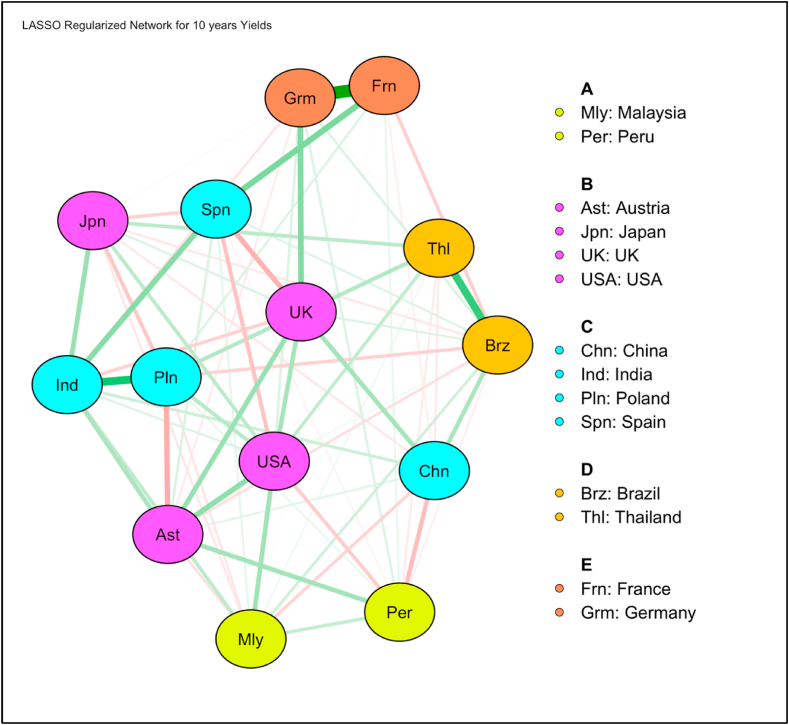
Estimated Network of Partial Correlations on 10 years Yields showing 5 communities.

**Fig. 12:**
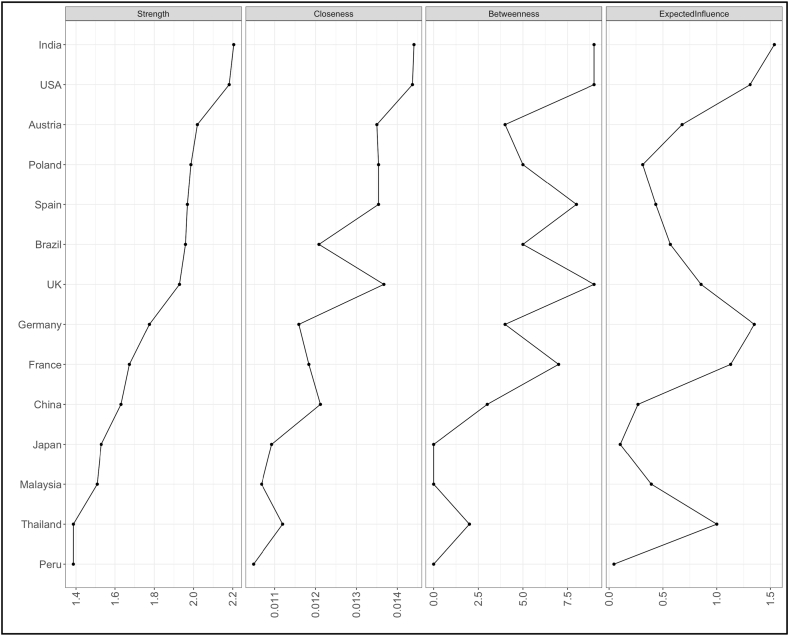
Centrality of 10 years yields network.

In Fig. 10, Fig. 12, centrality plots of 2- and 10- year yield networks are plotted using “raw” values of centrality measures like strength, betweenness, closeness and expected influence.

In 2-years yield network (Fig. 10), degree centrality or strength measure implicates that China is very connected node who can quickly connect with the wider network. In terms of betweenness and closeness, Poland is placed to influence the entire yield network. Since expected influence takes negative associations among nodes into account, USA found to be the strongest node. A high betweenness of Poland could indicate that this country holds authority over disparate clusters in this network. However, in 10-years yield network (Fig. 12), multiple nodes India, USA and UK found to have high betweenness. In terms of expected influence, India has highest value. India and USA both have high node centrality strength and expected influence values.

## 5 Robustness tests

In order to investigate empirically the statistical properties of the time series in the frequency domain a spectral analysis has been performed. It is known that most financial asset returns do not exhibit normal distribution (i.e. they have excess kurtosis indicating presence of heavy tails and/or non-zero skewness) but when these assets are measured on logarithmic scale, they are assumed to follow normal distribution – the lognormal distribution. Further, they show a large degree of variability in the time patterns and the autocorrelation function decrease to zero slowly and not exponentially. This persistence behavior of the asset prices is associated with the property of self-similarity. But this approach is not expected to follow pure power laws. Then we may use the power spectral density. We test our findings of persistence of bond yields using the Hurst exponents by applying the power spectral density plots. We compare the behavior of the power spectra *S(f)* with the function *f*^*−2H(2)−1*^ in the time-frequency domain analysis. The power spectral density is computed using the periodogram approach. We compute the slope of the power spectra for each cohort (reported in Table 7). The results are plotted in Appendix A.I.

**Table 7:** Slope (β) coefficients of the power spectra – *S(f)*$\propto f^{-\beta }$a.

Developed Countries	2-Year Yields	10-Year Yields
USA	−0.435	−0.514
UK	−0.446	−0.517
Germany	−0.442	−0.511
Japan	−0.485	−0.550
France	−0.451	−0.504
Austria	−0.451	−0.514
Spain	−0.524	−0.522
Poland	−0.353	−0.425
**Emerging Countries**		
India	−0.428	−0.469
China	−0.426	−0.418
Malaysia	−0.532	−0.479
Brazil	−0.426	−0.482
Peru	−0.476	−0.462
Thailand	−0.386	−0.475

To save space, we report the spectral density results for the USA, UK from cohort 1 and India, and China from Cohort 2 in Appendix A.I. The fitted values (curved) are compared with the classical *f*^*−5/3*^ power law shape over a range of frequencies (straight line). In all these plots, we find that the power law behavior holds for the bond yields well. The slope of the plot is the power spectra coefficient *β*, computed in equation (4), through a regression on a log-log scale. We can also see that the agreement between the power spectra behavior and the prediction from the generalized Hurst analysis is very satisfactory. We report the slopes of all the countries for the power spectra in Table 7 for comparison purpose. All the slopes are negative confirming to the agreement of the Hurst exponents of all the bond yields with the power spectra.

We have also computed the correlation stability (CS) coefficients (Table 8). The CS coefficients measures indicated that betweenness (CS(cor = 0.7) = 0.75), closeness (CS(cor = 0.7) = 0.75), and strength (CS(cor = 0.7) = 0.75) were stable and performed good. The cut-off for CS coefficients interpretability is 0.25 which concludes that orders of all the measures are interpretable.

**Table 8:** Centrality stability test values 2 years and 10 years yields.

Category	Betweenness (CS (cor = 0.7))	Closeness (CS (cor = 0.7))	Strength (CS (cor = 0.7))	Expected Influence (CS (cor = 0.7))
2 years Yields	0.75	0.75	0.75	0.75
10 years Yields	0.75	0.75	0.75	0.75

Stability analysis indicated that both the network structures were estimated accurately with small to moderate confidence intervals (Appendix A.II). The red line indicates the sample values, and the gray area are the bootstrapped Cis (confidence intervals). Each horizontal line represents the edge of the network, ordered from the highest edge-weight to lowest edge-weight. We also tested for significant differences in edge-weights and centralities using the bootstrapped difference test. From the resulting plots it can be seen almost all the edges are significantly different from one-another (Appendix A.II). We can see most of the node strengths are significantly different from each other. Node China has the largest strength for 2-year yields which is not significantly different from other nodes. Similar is applicable to nodes India and USA for 10-year yield (Appendix A.II).

## 6 Conclusion

In this paper, we study the scaling properties of the sovereign bond yields in its short (2-year) and long (10-year) legs using the generalized Hurst exponent. We provide evidence that fractal properties of the bond yields can be very well explained by the Hurst exponents and verified it using spectral analysis. The method provides an unbiased and efficient estimator which captures the multi-scaling properties of the yields. The method is then used to classify the markets for their persistent behavior. We find that in general, the GHE of the developed economies is less than those of the emerging economies indicating that bond yields in emerging economies are relatively more anti-persistent than those of the developed ones in both short and long legs of the yields. Further, using the network analysis approach, we detect community formation among the countries based on yields. Unlike the past studies (Sensoy et al., 2019), this study has used community detection algorithm to understand the clusters. Moreover, this study also focuses on interpreting the positive or negative correlations between nodes using partial correlation as the measure of edges. The networks of both the 10-year and 2-year yields indicate communities’ formations of various countries which result in providing diversification benefits to the investors. Though in the case of long tenure bonds, most of the emerging countries are classified into one community, this concentration is more evident in the short tenure bonds. This suggests that while diversification can be achieved in 10-year bonds, they are less prominent in the 2-year bonds. Our methodology may be used for portfolio diversification using sovereign bonds as this can help in evaluating the risk of big losses or gains as a function of time.

The study has several implications for managers and policy makers. Our study uses a novel and robust methodology to investigate the long-term dependencies in the sovereign bond yields. Portfolio managers and investors who look to diversify their portfolio in risk free securities can use the results for achieving higher returns on their investments. Further, the dynamic relationship among the sovereign yields makes it important for the policy makers to design their monetary policies keeping in mind the spillover of the yields among various markets.

## 7 Limitations of the study

Although we use a finite sample of select developed and emerging countries for our study, we feel that there is a necessity to include other countries especially from emerging economies to better understand the dynamics of dependencies and correlation among the bond yields. There is widespread heterogeneity in the policy environments among various countries which might affect the behavior of these bonds. Therefore, future research can use our methodologies to investigate how the country specific variables affect the correlation and time series dynamics of the sovereign bonds. Further, many studies in the recent years have shown that a systemic crisis like Covid-19 pandemic can have significant impact on the relationship among the asset prices and we therefore recommend that future research may be taken up to study the correlation among the bonds considering such crisis periods. Further, the network analysis can be extended over a specific period of post-covid analysis and can be compared with pre-covid network to understand the difference in trend and relationships which most of the researchers have found for stock markets [49]. This would give a clear perspective on requirement of policy changes for portfolio diversification in case of global crisis like COVID – 19.

## Author contribution statement

Santanu Das: Aritra Pan: Conceived and designed the experiments; Performed the experiments; Analyzed and interpreted the data; Contributed reagents, materials, analysis tools or data; Wrote the paper.

Nikunj Kumar Jain: Analyzed and interpreted the data; Contributed reagents, materials, analysis tools or data; Wrote the paper.

## Data availability statement

Data will be made available on request.

## Additional information

Supplementary content related to this article has been publish online at [URL].

## Advisory/management and consulting positions

The author(s) declare that one of the co-authors – Prof Nikunj Kumar Jain is Associate Editor at Heliyon Journal (Business and Economics section).

## Funding

Not applicable.

## Declaration of competing interest

The authors declare that they have no known competing financial interests or personal relationships that could have appeared to influence the work reported in this paper.

## Figures and Tables

**Figure:**
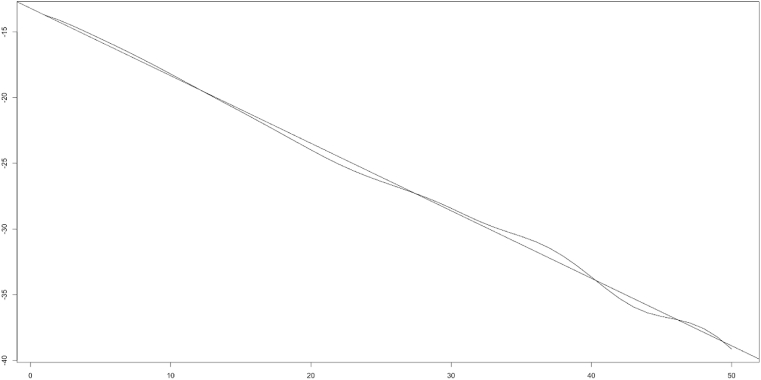


**Figure:**
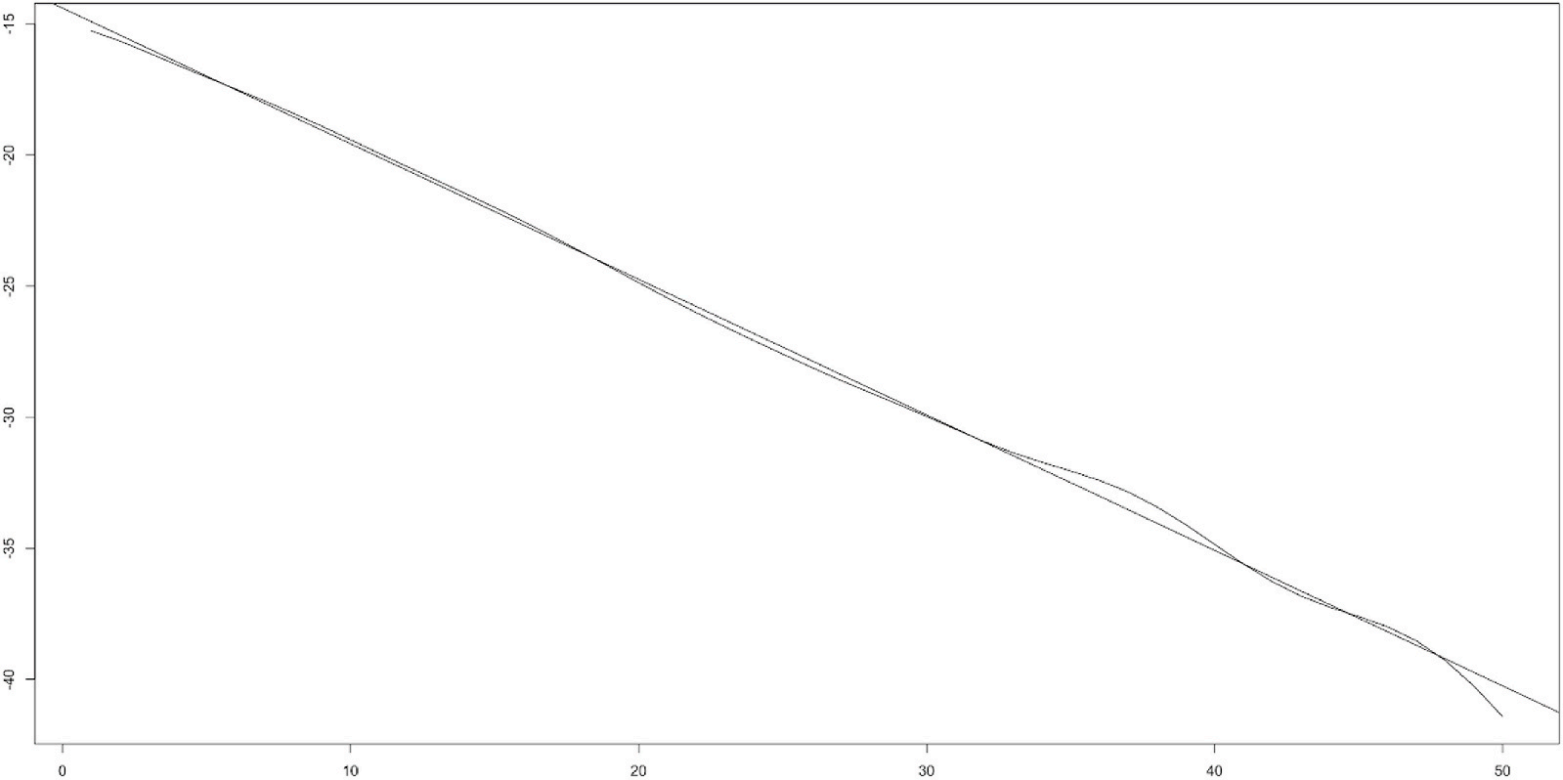


**Figure:**
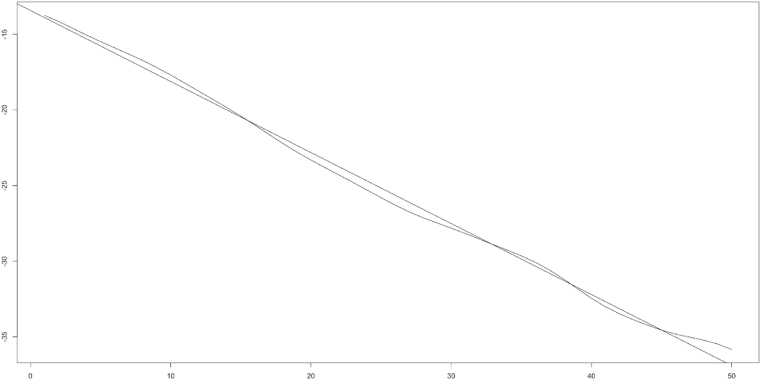


**Figure:**
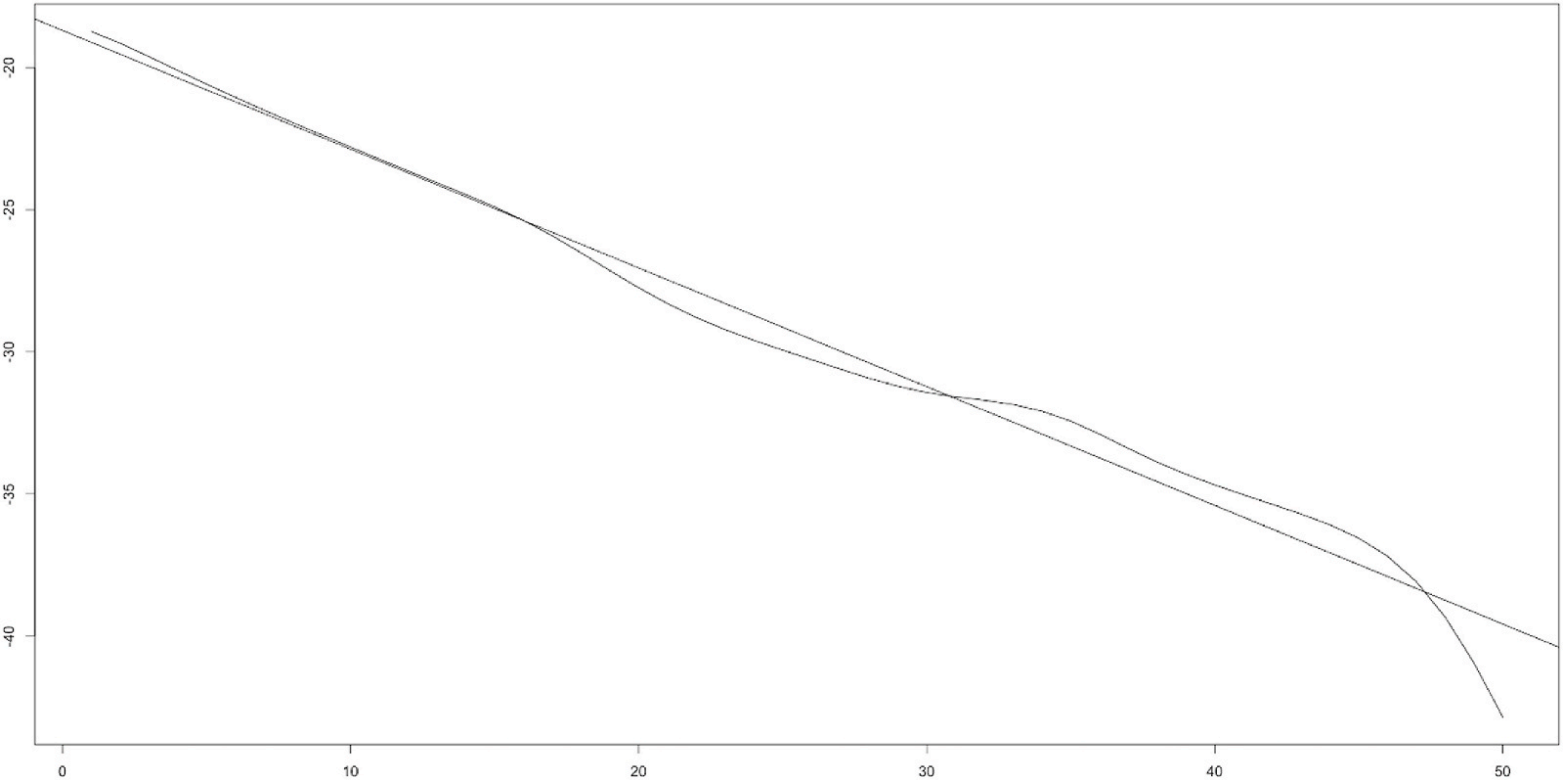


**Figure:**
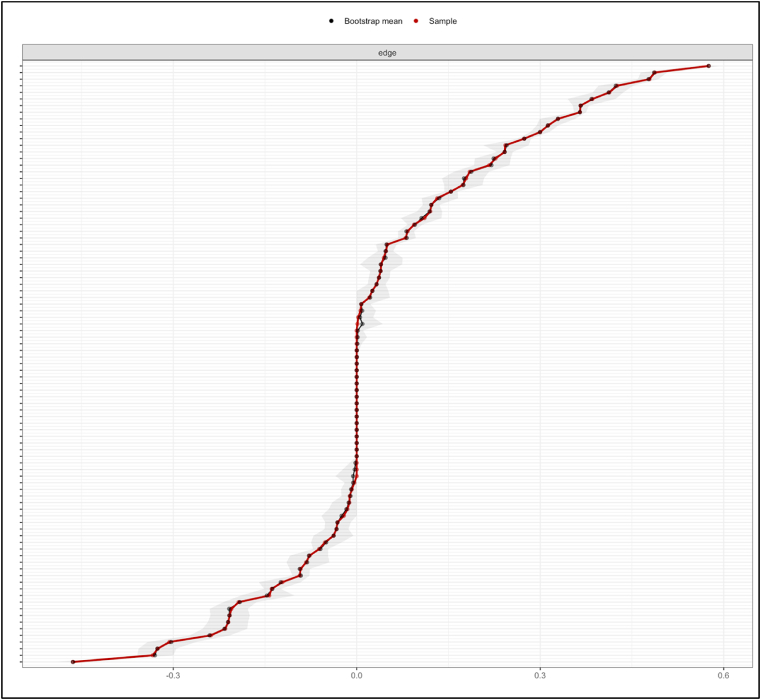


**Figure:**
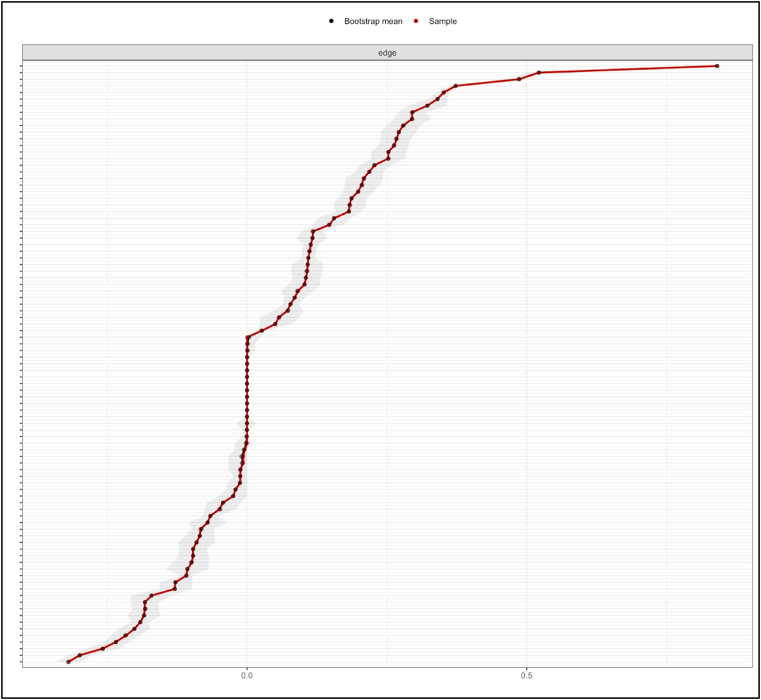


**Figure:**
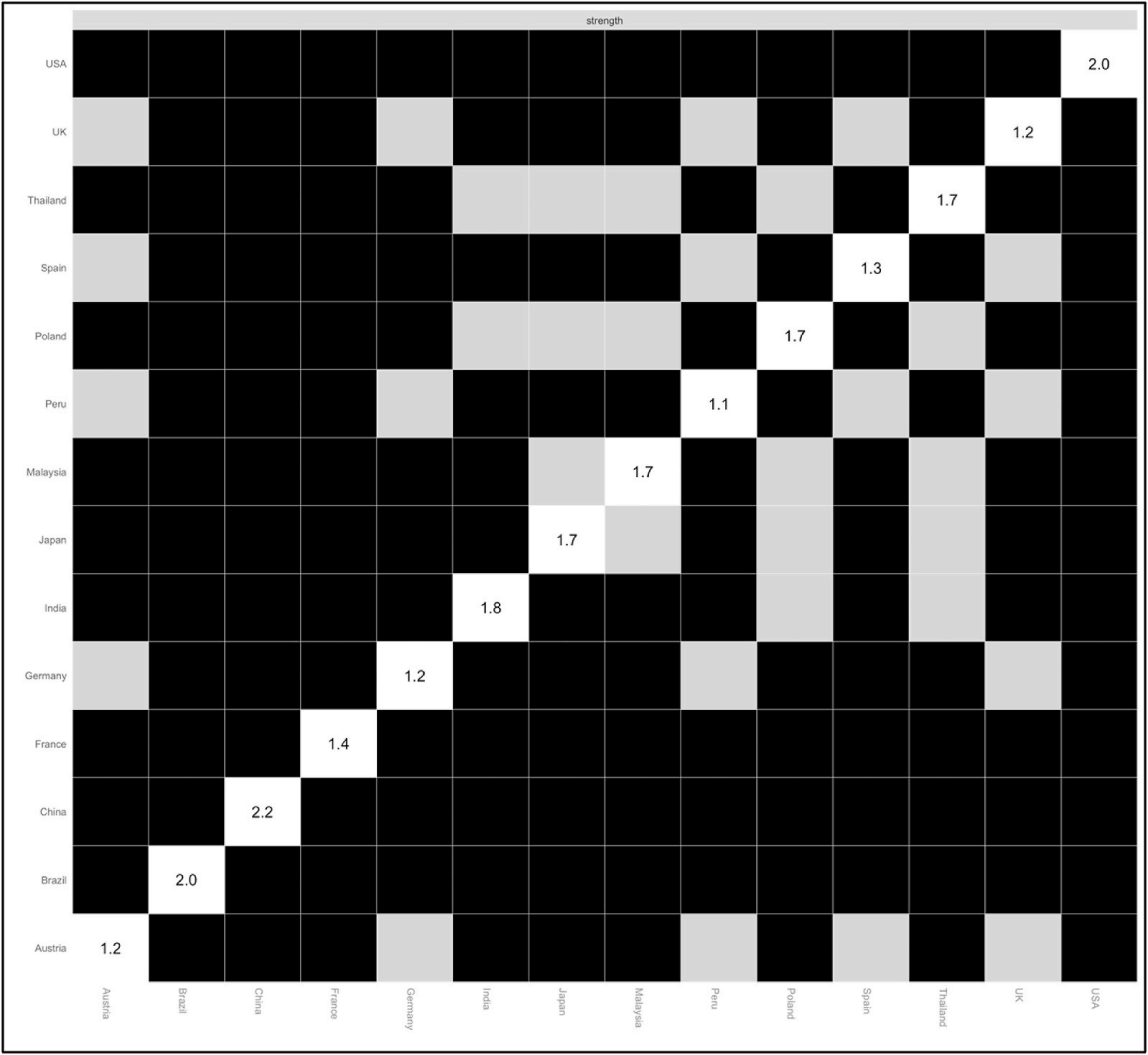


**Figure:**
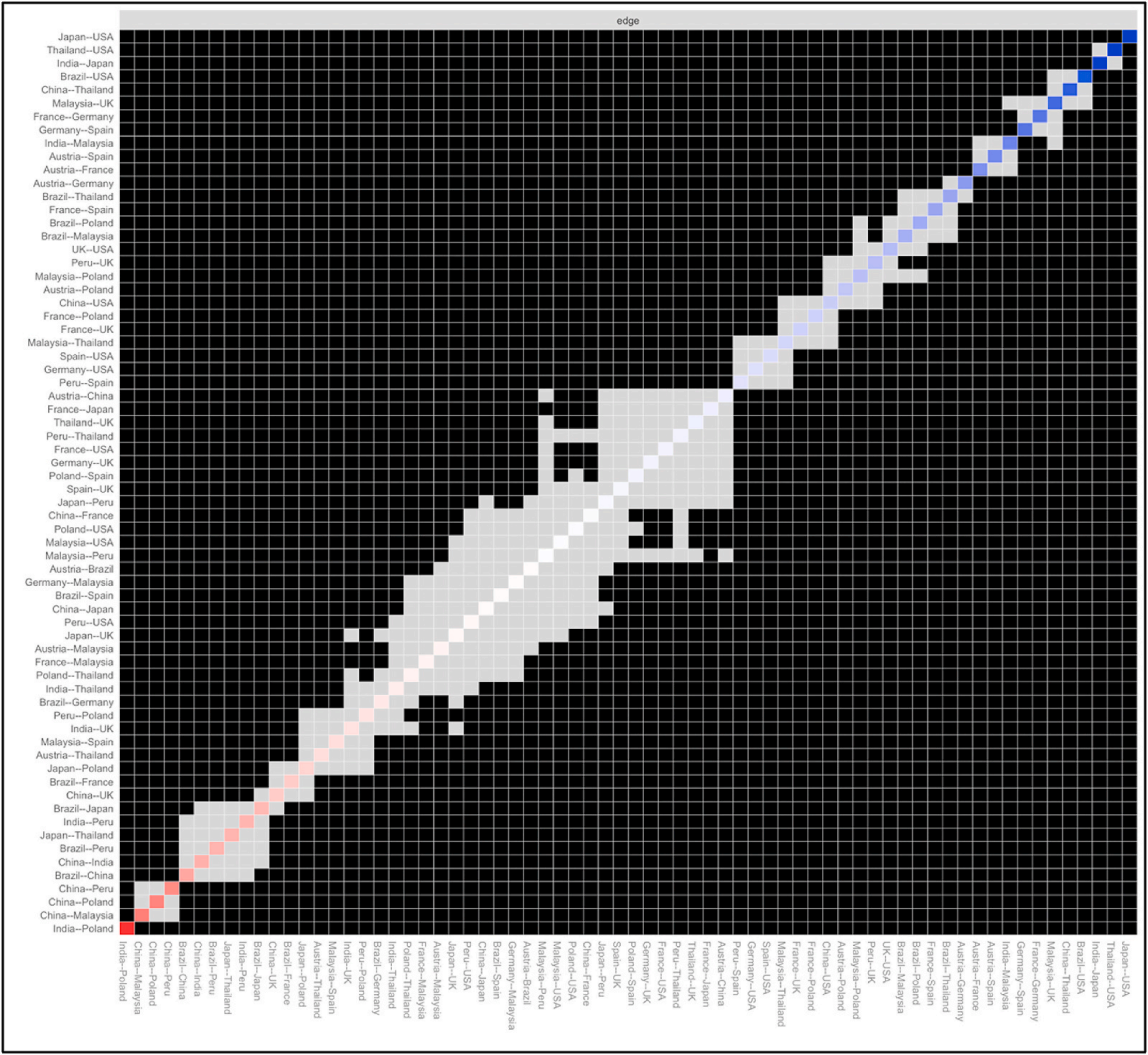


**Figure:**
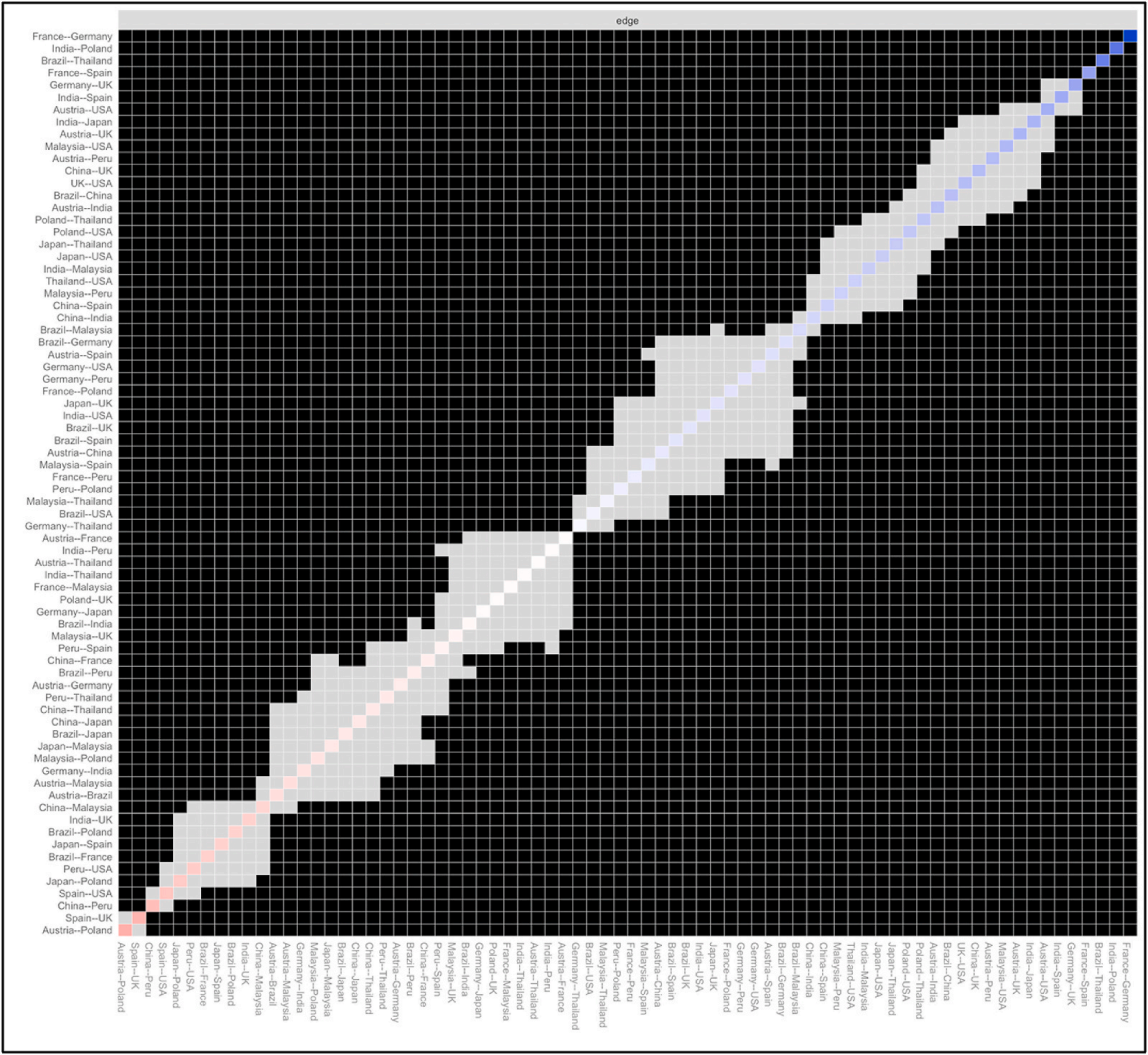


**Figure:**
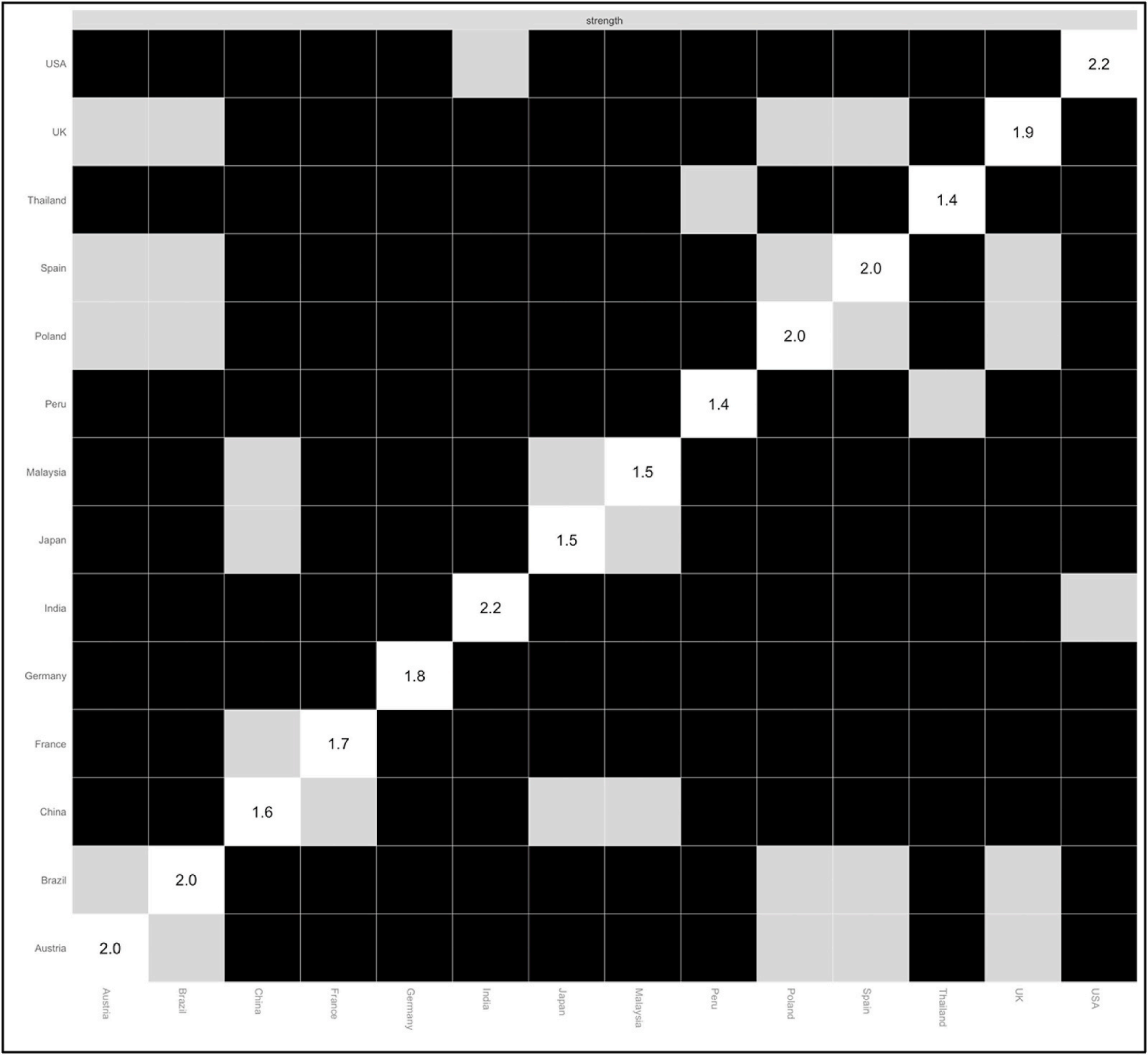


## Appendix

### A.I - Spectral Density Plots

Spectral Density plots of (10-year Sovereign bond yields) in USA – X-axis represents log (frequency) and Y-axis represents log (Spectral Density).

Spectral Density plots of (10-year Sovereign bond yields) in UK – X-axis represents log (frequency) and Y-axis represents log (Spectral Density).

Spectral Density plots of (10-year Sovereign bond yields) in India – X-axis represents log (frequency) and Y-axis represents log (Spectral Density).

Spectral Density plots of (10-year Sovereign bond yields) in China – X-axis represents log (frequency) and Y-axis represents log (Spectral Density).

### A.II – Robustness Check of Network Models

Bootstrapped confidence intervals of estimated edge-weights in 2 years Yields Network.

Significant differences in edge-weights in 2 years Yields Network.

Significant differences in node strength in 2 years Yields Network.

Bootstrapped confidence intervals of estimated edge-weights in 10 years Yields Network.

Significant differences in edge-weights in 10 years Yields Network.

Significant differences in node strength in 10 years Yields Network.
